# Acetate Combined with CO_2_ as Effective Carbon Sources for the Production of Resistant Starch in a Marine Microalga *Tetraselmis subcordiformis*

**DOI:** 10.3390/foods14112004

**Published:** 2025-06-05

**Authors:** Haoyu Zhang, Yuhan Shen, Yufei Liu, Xiuyuan Ran, Yongkui Zhang, Jing Chen, Changhong Yao

**Affiliations:** Department of Pharmaceutical & Biological Engineering, School of Chemical Engineering, Sichuan University, Chengdu 610065, China; hyzhang2025@163.com (H.Z.); 2024323070034@stu.scu.edu.cn (Y.S.); 2023223070097@stu.scu.edu.cn (Y.L.); 2023323070036@stu.scu.edu.cn (X.R.); zhangyongkui@scu.edu.cn (Y.Z.); jing.chen@scu.edu.cn (J.C.)

**Keywords:** microalgae, mixotrophy, amylose, amylopectin, enzyme activity, morphology, ordered structure, digestibility

## Abstract

Microalgae are considered as sustainable starch producers, yet the carbon sources for this process in terms of starch productivity and functionality require further elucidation. The present study investigated the roles of CO_2_ and acetate on the starch production in a marine microalga *Tetraselmis subcordiformis*, and the ordered structure and digestibility of the starches obtained were characterized. CO_2_ and acetate could serve as efficient carbon sources for *T. subcordiformis* to accumulate starch, with the maximum starch content, yield, and productivity reaching 66.0%, 2.16 g/L, and 0.71 g/L/day on day 3 and the maximum biomass productivity reaching 0.94 g/L/day on day 2, respectively, when 2.5 g/L sodium acetate and 2% CO_2_ were simultaneously applied. The addition of acetate under 2% CO_2_ improved the photosynthetic efficiency and enhanced the activity of ADP-glucose pyrophosphorylase, facilitating the biomass and starch production. The supply of CO_2_ and acetate changed the amylose/amylopectin ratio by affecting the activity of starch branching enzymes and isoamylases. FTIR and XRD analyzes showed that the supply of CO_2_ reduced the long- and short-range ordered structure of starch, while acetate promoted the production of additional B- and V-type starch, resulting in a reduced digestibility. The combined supply of 2% CO_2_ and 5 g/L sodium acetate enabled the most efficient production of functional resistant starch (RS) measured with Englyst’s method, with a maximum RS content and yield reaching 13.7%DW and 0.40 g/L, respectively, on day 3. This study provided novel insights into the efficient production of high value-added functional starch (RS) from microalgae.

## 1. Introduction

Starch is a polysaccharide composed of glucose monomers. It is an important energy reserve for plants and algae, and is also the basic food source for humans [1]. Starch can be generally classified into two types according to the polymerization structure of the glucose monomers: amylose (Am) and amylopectin (Ap). Am is a linear glucan connected by α-(1,4)-glycosidic bonds, usually accounting for about 15–30% of the total starch [2]. Amylopectin (Ap), which makes up 70–85% of the total starch, has a branched glucan chain linked by α-(1,6)-glycosidic bonds in addition to the linear α-(1,4)-glucan chain as present in Am [3]. Starch is biosynthesized and stored as semicrystalline granules. There are three types of starch known as A-, B-, and C-type based on their XRD (X-ray diffraction) patterns, among which C-type starch is a mixture of A- and B-type allomorphs within the same granule [4]. In addition, a V-type starch which describes the co-crystallization of single helices of amylose with iodine, DMSO, alcohol, and fatty acids is also present [4]. The digestibility of starch determines its downstream application. For nutritional purposes, starch in foods is classified into rapidly digestible starch (RDS), slowly digestible starch (SDS), and resistant starch (RS) [5]. Among them, resistant starch (RS) represents a special functional starch that possesses a number of significant biological effects such as improving intestinal health, facilitating glycemic balance, adjusting lipid metabolism, and helping with energy/weight management, which therefore has attracted widespread attention as healthy and functional food ingredients [6,7].

Currently, natural starch is mainly produced from crops such as corn, wheat, rice, and cassava [8], which have generally suffered from uncontrollable cultivations with a large demand for agricultural resources (e.g., arable land, fresh water, fertilizers, and pesticides) that could be unsustainable [9,10,11]. Microalgae have been considered as efficient and sustainable starch producers in recent years [12,13]. Compared with terrestrial plants, microalgae have the unique advantages of a high photosynthetic efficiency, short growth cycle, potent carbon sequestration ability, and a controllable cultivation environment, as well as being free from seasonal and geographic restrictions, while having the advantages of saving fresh water resources (especially marine microalgae), and not competing with humans for food and land [14,15,16,17]. Microalgae can accumulate starch in large quantities under certain conditions. For example, *Chlorella vulgaris*, *Chlamydomonas reinhardtii*, and *Tetraselmis subcordiformis* can accumulate 38–69% of starch in dry biomass under high light coupled with nutrient-limitation stress [18,19,20,21], which highlights the potential of these microalgae as excellent sources of sustainable green starch in the future. In general, starch from microalgae shares similar components (Am and Ap) and crystalline structures (A type) with cereal crops such as corn and rice, but the morphology and fine structures are largely different, which makes them differ in digestibility as well [10,22,23,24]. Moreover, for the past two years, it has been proven in *Chlorella* sp., *Chlorella sorokiniana*, and *Tetraselmis subcordiformis* that microalgae can synthesize RS with varied contents [10,25,26,27]. However, the research on the production of this kind of functional starch from microalgae is still in its infancy, which needs systematic and in-depth research to improve the production efficiency.

The carbon source has significant impacts on microalgae biomass production [28]. The use of inorganic carbon sources, typically CO_2_, can not only improve the efficiency of microalgae biomass and starch production [28], but also affects the composition, structure, and granule morphology of the synthesized starch [23,29]. Acetate is an efficient organic carbon source for many microalgae, the use of which is an important strategy to improve the economy of microalgae biomass production [30]. Acetate can be converted into acetyl-CoA in microalgae cells, promoting the glyoxylate cycle and TCA cycle, thereby increasing the biomass yield [31]. It was shown that adding an appropriate amount of acetate for microalgae as a carbon source in a mixotrophic cultivation mode can promote cellular respiration [31,32] while increasing the RuBisCO activity [33] and photosynthetic pigment content [34,35], which significantly increased microalgae biomass production [31]. With the maturing of advanced acetate synthesis technologies which coupled with CO_2_ emission reduction and waste treatment (such as artificial photosynthesis, microbial electrosynthesis, C1 gas fermentation, etc.), the use of acetate as a renewable, cheap, and efficient organic carbon source to cultivate microalgae for biomass production has excellent prospects [30,36]. At present, most studies focus on the effects of acetate on microalgae biomass production and biochemical composition under CO_2_ deficiency (air condition, ~0.04% CO_2_) [35,37], and there are few reports on the combined regulation of microalgae biomass production by CO_2_ and acetate. In addition, the effects of the acetate supply under different CO_2_ availabilities on the composition (Am/Ap ratio), granule morphology, ordered structure, and digestibility characteristics of microalgal starch (such as the RS content) have not been explored, although these properties could largely determine the downstream application of microalgal starch.

This study aims to develop a strategy for the efficient production of functional starch from the marine microalga *Tetraselmis subcordiformis* using inorganic (CO_2_) and organic carbon (acetate) sources. The effects of CO_2_ and acetate on the growth, photosynthetic efficiency, biomass production, and biochemical composition of *Tetraselmis subcordiformis* under N-deficiency conditions were studied in detail, with a special focus on the improvement of starch yield and quality as well as the underlying basis involved at the enzyme activity level. In addition, the granule morphology, ordered structure (including long- and short-range ordered structures), and digestion characteristics of microalgal starch produced under the above conditions were also determined, based on which the RS production efficiency was then evaluated.

## 2. Materials and Methods

### 2.1. Algal Strain and Culture Conditions

*Tetraselmis subcordiformis* FACHB-1751, obtained from the Marine Bioengineering Group of the Dalian Institute of Chemical Physics, Chinese Academy of Sciences, was previously cultured in artificial seawater (ASW) containing 27 g/L NaCl, 6.8 g/L MgSO_4_·7H_2_O, 5.6 g/L MgCl_2_·6H_2_O, 1.5 g/L CaCl_2_·2H_2_O, 0.82 g/L KCl, 1.3 mg/L FeCl_3_·6H_2_O, 0.36 mg/L MnCl_2_·4H_2_O, 33.6 mg/L H_3_BO_3_, 45.0 mg/L EDTA-Na_2_, 0.21 mg/L ZnCl_2_, 0.2 mg/L CoCl_2_·6H_2_O, 0.09 mg/L (NH_4_)_4_Mo_7_O_24_·4H_2_O, and 0.137 mg/L CuCl_2_·2H_2_O with the addition of 1.113 g/L KNO_3_, 0.067 g/L KH_2_PO_4_, 0.81 g/L Tris, and 0.33 mL/L glacial acetic acid [18]. Algae cells were collected in the late exponential phase and washed twice with nitrogen-free artificial seawater (ASW-N) in which nitrate was removed.

The algal cells were then inoculated into ASW-N medium with the addition of 0.067 g/L KH_2_PO_4_ at an initial optical density (OD_750_) of 0.750, with sodium acetate added to final concentrations of 0, 2.5, 5, and 10 g/L (0, 31, 62, and 124 mM acetate, respectively) based on previous research [38]. The microalgae cells were cultured under photoautotrophic conditions in a cylindrical glass bubble-column photobioreactor (diameter of 48 mm and height of 550 mm) with a working volume of 600 mL, and the cultivation temperature was maintained at 25 ± 2 °C. Air (0.04% CO_2_) or 2% CO_2_-enriched air that usually offers optimal microalgal growth [39] was continuously sparged into the medium at a rate of 0.4 vvm. All the experimental treatments are summarized in Table 1. A continuous illumination was provided by a cold, white, fluorescent lamp from one side, delivering a photosynthetic photon flux density (PPFD) of 180 μmol·m^−2^·s^−1^. No extra pH control strategy was applied to test the feasibility of the formation of the auto-buffering system upon carbon source addition. All experiments were performed in triplicate.

### 2.2. pH and Growth Measurement

The pH was measured using a standard benchtop pH meter (ARK, pHS-4C+, Chengdu, China). Cell growth was determined by measuring the optical density at 750 nm (OD_750_) using a UV-Vis spectrophotometer (AOE, U*V*/*V* is A-360, Shanghai, China) after appropriate dilution [40]. Cell dry weight (DW) was determined gravimetrically as described previously [40] and the biomass productivity (*P_b_*, g/L/day) was calculated using the following formula:

(1) $$P_{b}=\left(DW_{t}-DW_{0}\right)/t$$

where $DW_{t}$ and $DW_{0}$ represent the cell dry weight at time *t* and the initial time (0), respectively.

### 2.3. Photosynthetic Performance Analysis

The photosynthetic performance of the microalgae was measured using a chlorophyll fluorometer (Os30p+, Optisciences, Hudson, NH, USA) and calculated according to the methods described by Strasser and Srivastava [41].

### 2.4. Starch Measurement

The starch in the microalgal biomass was extracted with 45% HClO_4_ following the previous method [10]. Briefly, 0.5 mL of 45% HCIO_4_ was added to the algal sludge, and the mixture was shaken for 10 s before letting it stand for 4 min. Subsequently, 6 mL of deionized water was added to the extracts, followed by centrifugation at 10,000 r/min for 5 min, and the supernatant was then collected. The sample was mixed with an equal volume of 0.01 N I_2_-KI. After incubation at room temperature for 15 min, the mixture was measured colorimetrically at 618 nm and 550 nm where the absorption maxima for amylose and amylopectin occurred, respectively [42]. The concentrations of amylose (*Am*) and amylopectin (*Ap*) were calculated according to the method described by Landers et al. [43] as follows:

(2) $$Am=\left(A_{618}\times b_{2}-A_{550}\times b_{1}\right)/\left(a_{1}\times b_{2}-a_{2}\times b_{1}\right)$$

(3) $$Ap=\left(A_{550}\times a_{1}-A_{618}\times a_{2}\right)/\left(a_{1}\times b_{2}-a_{2}\times b_{1}\right)$$

where *A*_618_ and *A*_550_ represented the absorbance at 618 nm and 550 nm, respectively. *a*_1_ and *a*_2_ were the absorptivity values of amylose at 618 nm and 550 nm, respectively, and *b*_1_ and *b*_2_ were the absorptivity values of amylopectin at 618 nm and 550 nm, respectively. The concentrations of the total starch (*C_s_*) was the sum of *Am* and *Ap*.

The starch content (*ω_s_*, %DW) and starch productivity (*P_s_*, g/L/day) were calculated using the following formulas:

(4) $$\omega_{s}=C_{st}/DW_{t}\times 100\%$$

(5) $$P_{s}=\left(C_{st}-C_{s0}\right)/t$$

where $C_{st}$ (g/L) and $C_{s0}$ (g/L) represent the concentrations of the total starch at time *t* and the initial time (0), respectively.

### 2.5. Biochemical Component Analysis

The total protein was extracted with 0.5 M NaOH at 80 °C for 10 min and measured following a BCA method (BCA Protein Assay Kit, Biotechnology, Shanghai, China). The total lipid content was analyzed with a sulfo-phospho-vanillin (SPV) colorimetric method as described in [44]. Briefly, 0.1 mL of deionized water and 1 mL of H_2_SO_4_ were added to the algae sludge. After boiling for 10 min and cooling in ice for 5 min, 2.4 mL of PV solution (vanillin: phosphoric acid = 0.6:400, *w*/*v*) was added before shaking for 20 min. The lipid content was calculated based on the absorbance at 530 nm using rapeseed oil as the standard for calibration.

### 2.6. Acetate Analysis

The acetate concentration was analyzed using a gas chromatograph (GC9720Plus, Fuli, Taizhou, China) as described by Lin et al. [45]. The supernatant was mixed with an equal volume of 500 mg/L 4-methylvaleric acid, acidified with 100 µL of formic acid, and ultrasonicated for 5 min. The sample was then injected into a capillary RB-FFAP column (30 m × 0.5 µm × 0.32 mm, Fuli, Taizhou, China) with a flame ionization detector (FID). Argon was used as a carrier gas with a flow rate of 25 mL/min, and the injection and detection temperatures were set at 250 °C. The acetate concentration was calculated based on the peak area ratio of acetate to 4-methylvaleric acid.

### 2.7. Cell Morphology

The cell morphology and starch accumulation were observed by staining the algal cells with an equal volume of iodine solution (0.2% I_2_, 2% KI) followed by examining the samples under an optical microscope (BMC513-IPL, Shanghai, China).

### 2.8. Enzyme Activity Assays

Algal crude extracts were prepared as described previously [46]. Algae with a volume of 40 mL were harvested by centrifugation at 10,000× *g* for 5 min at 4 °C, and the cells were homogenized by ultrasonication in the extracting buffer (50 mM Tris HCl, pH = 7.5; 2 mM DTT; 2 mM EDTA-Na_2_). The crude extracts were obtained by centrifugation at 6500× *g* for 10 min at 4 °C. The protein concentration in the crude extract was quantified with the Bradford method. The ADP-glucose pyrophosphorylase activity was measured using the Adenosine diphosphate glucose phosphorylase (AGPase) activity detection kit (Solarbio, Beijing, China). In principle, the reverse reaction was determined with the detection of glucose-1-phosphate formation which coupled to an increase in the NADH concentration measured at 340 nm [40]. The branching enzyme and isoamylase activities were analyzed by zymogram according to the methods described previously [40]. Briefly, a native resolving gel consisting of 7.5% (*w*/*v*) acrylamide and 0.3% (*w*/*v*) potato tuber amylopectin with the stacking gel containing 3.3% (*w*/*v*) acrylamide was used for the electrophoresis. Electrophoresis was performed in an ice-water bath at 8 mA for 0.7 h, followed by electrophoresis at 15 mA for 2.5 h. After electrophoresis, the gel was rinsed twice with the reaction solution (50 mM Citrate-Na_2_HPO_4_, pH = 6.0; 2 mM DTT) and then imbibed with 20 mL of the same solution at room temperature for 2 h under a constant shaking of 80 r/min. Branching enzyme and isoamylase activities were visualized as brown and blue bands in the gel, respectively, after incubation in the iodine solution (0.1% I_2_, 1% KI) for 2 min.

### 2.9. Starch Isolation and the In Vitro Digestion of Starch

Starch was extracted from lyophilized algal powder using a modified method from Buleon et al. [47]. Briefly, the lyophilized powder was resuspended in TE buffer (50 mM Tris-Cl, 2 mM EDTA, pH 7.5) at a concentration of 50 mg/mL. The suspension was ultrasonicated in an ice-water bath at 200 W for 15 min [22] and centrifuged at 10,000 r/min for 20 min at 4 °C to obtain crude starch. The pellet was resuspended in TE buffer and then passed twice through a Percoll (Solarbio)/85% NaCl solution (9:1, *v*/*v*) with 4.0 mL of Percoll/85% NaCl solution per 1.0 mL of crude starch suspension [10]. The supernatant was discarded, and the pellet was washed twice with deionized water followed by absolute ethanol. Finally, the starch was freeze-dried for 48 h and ground into a fine powder.

The total starch content (TS) was determined using a Total Starch (AA/AMG) Assay Kit (K-TSTA-100A, Megazyme International Ireland Ltd., Bray, County Wicklow, Ireland) following the manufacturer’s instructions. A total of 200 μL of 1.7 mol/L NaOH was added to a 10 mg starch sample, followed by a thorough mix and stirring in an ice-water bath for 15 min. A total of 800 μL of acetic acid buffer (600 mM, pH = 3.8), 100 μL of α- amylase (300 U/mL), and 10 μL of amyloglucosidase (3300 U/mL) were added in sequence before shaking thoroughly and incubation at 50 °C for 30 min. The reaction mixture was then centrifuged at 13,000 r/min for 5 min, and the glucose content in the supernatant was measured using the GOPOD method.

For in vitro digestibility, a modified method based on Englyst and Cummings [48] was used. A total of 10 mg of starch was suspended in 200 µL of 100 mM acetate buffer (pH 5.2) and heated at 95 °C for 30 min. After cooling to 37 °C, the mixture was incubated with an enzyme complex (α-amylase and AMG) and aliquots were taken at 20 and 120 min, respectively. The glucose content was determined using GOPOD, and rapidly digestible starch (*RDS*), slowly digestible starch (*SDS*), and resistant starch (*RS*) were calculated using the following formulas:

(6) $$DS=G_{20}\times 0.9/TS\times 100\%$$

(7) $$SDS=\left(G_{120}-G_{20}\right)\times 0.9/TS\times 100\%$$

(8) RS=TS−RDS−SDS

where $G_{20}$ and $G_{120}$ represent the glucose content at 20 min and 120 min, respectively, and *TS* represents the total starch content.

### 2.10. Scanning Electron Microscopy (SEM)

The morphology of starch granules was observed using a scanning electron microscope (HITACHI S-4800, Hitachi, Tokyo, Japan). Starch samples were fixed on the specimen stage using conductive double-sided tape and sputter-coated with gold to ensure conductivity. All observations were performed under vacuum conditions at an accelerating voltage of 10 kV and a magnification of 50,000×.

### 2.11. X-Ray Diffraction (XRD)

The crystalline structure analysis of starch was performed using an X-ray diffractometer (Empyrean, Panaco, The Netherlands). Samples were scanned over a 2θ range of 5–40° with a step size of 0.02° using Cu–Kα radiation as the X-ray source. The relative crystallinity (RC) of starch was calculated as the ratio of the crystalline component to the total diffraction area, as estimated using MDI Jade 6.0 software.

### 2.12. Fourier Transform Infrared Spectroscopy (FTIR)

FTIR spectra of the starch samples were recorded using a Nicolet iS50 FTIR spectrometer (Thermo Scientific, Madison, WI, USA) at a resolution of 4 cm^−1^ over the range of 400–4000 cm^−1^. The obtained spectra were analyzed using OMNIC 9.0 (Visition9.0, Thermo Scientific, USA). The intensity ratio at 1047 cm^−1^/1022 cm^−1^ (R_1047/1022_) reflects the degree of short-range ordered structure in the starch [10].

### 2.13. Statistical Analysis

All experiments were performed in triplicate, and the data were analyzed using IBM SPSS Statistics 30.0 software (license: EEA239D6ISWSCSYYAPL6CLGQ3Z24UGTJASFW 3PVPUBXVAJSFZC8LGOQ5DMSK4W6VGHCXQJE7ZWGECT6YPJUO7ZPJX2DXIEVO 5NM). The results are presented as the mean values ± SD of three independent experiments. The Shapiro–Wilk Test and Levene Test were used to check the normality and homoscedasticity of the data, respectively. One-way analysis of variance (ANOVA) was used for parameters that conform to the normality and homogeneity of variance, before which Tukey’s HSD post-hoc test was applied for multiple comparisons; otherwise, the Kruskal–Wallis test was used followed by Dunn test as a post-hoc test. In all cases, data were considered significantly different when *p* < 0.05.

## 3. Results and Discussion

### 3.1. Production of Starch with Different Carbon Sources

To assess the effects of CO_2_ and acetate on the starch production in *Tetraselmis subcordiformis*, acetate (Ac) with final concentrations of 0, 2.5, 5, and 10 g/L were added to the N-deficient culture systems with air (Air-Ac-0, Air-Ac-2.5, Air-Ac-5, and Air-Ac-10) or 2% CO_2_-enriched air (CO_2_-Ac-0, CO_2_-Ac-2.5, CO_2_-Ac-5, and CO_2_-Ac-10) supplied to the cultures, respectively.

#### 3.1.1. Cell Growth and Biomass Production

**Figure 1:**
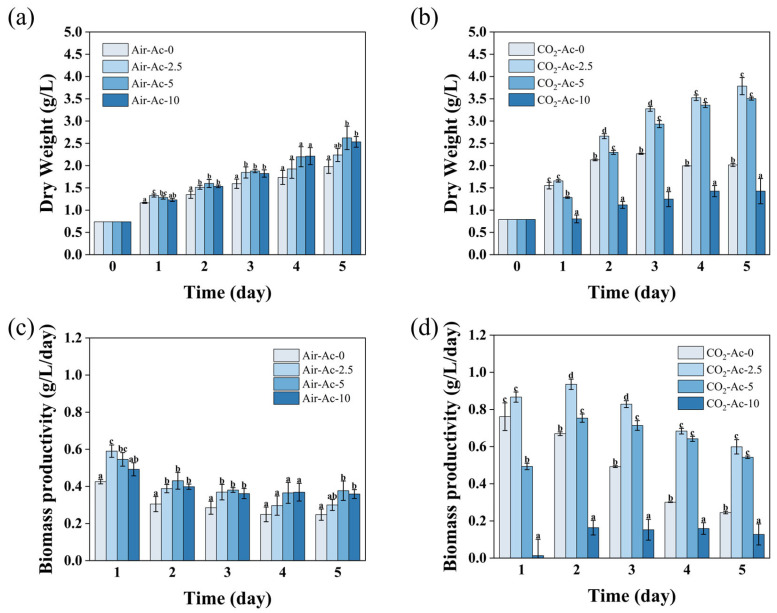
The biomass production (**a**,**b**) and biomass productivity (**c**,**d**) in *Tetraselmis subcordiformis* under different concentrations of acetate (0 g/L, 2.5 g/L, 5 g/L, and 10 g/L) conditions with air (**a**,**c**) or 2% CO_2_ (**b**,**d**) aerations during the N-deficient cultivation process. The different letters above the column in the same cultivation day represent the significant difference (*p* < 0.05) among various cultivation conditions.

Without additional carbon sources (Air-Ac-0), microalgae could utilize the limited CO_2_ (~0.04%) in air to slowly grow (Figure S1a) and accumulate biomass (Figure 1a,c), and the dry weight of the cells increased from 0.74 g/L on day 0 to 1.98 g/L on day 5. The addition of acetate significantly promoted the growth of the cells. The addition of 2.5–5 g/L of acetate (Air-Ac-2.5 and Air-Ac-5) resulted in a 30.1–40.6% increase in cell growth (OD_750_) over 5 days compared with the control (Air-Ac-0) (Figure S1a). The dry weight of the cells reached 2.24–2.62 g/L in the cultures of Air-Ac-2.5 and Air-Ac-5, representing a 13.1–32.3% increase compared with the control (Air-Ac-0), and the elevation was concentration-dependent (Figure 1a). Further increasing the acetate concentration to 10 g/L (Air-Ac-10) could not further improve the cell growth and biomass accumulation (Figure S1a and Figure 1a). Accordingly, biomass productivity was enhanced by 25.5~32.9% in Air-Ac-2.5~Air-Ac-10 compared to Air-Ac-0, reaching 0.30~0.32 g/L/day on day 5 (Figure 1c).

**Figure 2:**
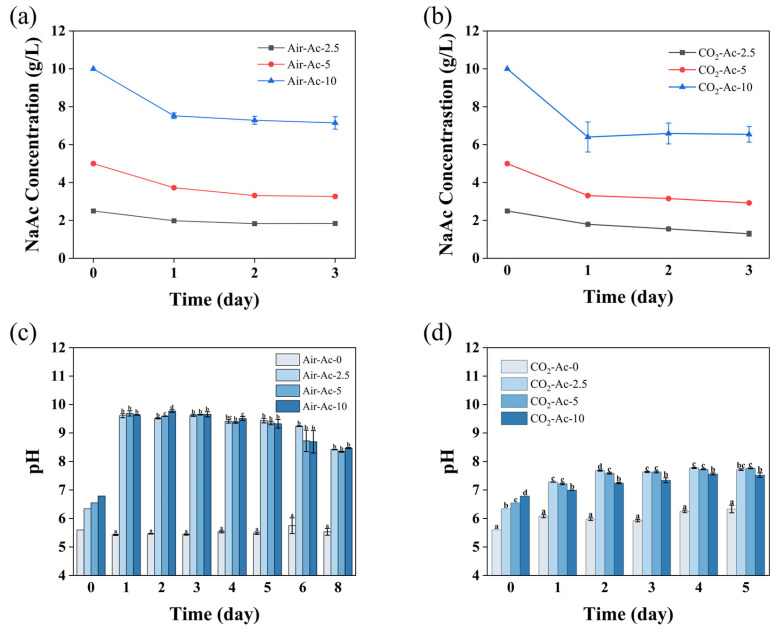
Acetate concentrations in the medium (**a**,**b**) and pH variations (**c**,**d**) in *Tetraselmis subcordiformis* under different concentrations of acetate (0 g/L, 2.5 g/L, 5 g/L, and 10 g/L) conditions with air (**a**,**c**) or 2% CO_2_ (**b**,**d**) aerations during the N-deficient cultivation process. The different letters above the column in the same cultivation day represent the significant difference (*p* < 0.05) among various cultivation conditions.

An acetate analysis (Figure 2a) showed that the decline of the acetate concentration in the medium occurred mainly on day 1, and the decreasing rate increased with the increase of acetate addition. This implied that acetate could be assimilated by the microalgae. Without the addition of additional carbon sources (Air-Ac-0), the pH of the medium was stable between 5.4 and 5.8 (Figure 2c), whereas the pH increased from 6.3–6.8 to 9.6–9.7 on the first day and then remained essentially constant with the addition of 2.5–10 g/L of acetate (Air-Ac-2.5–Air-Ac-10). The increase of pH was basically positively correlated with acetate assimilation (Figure 2a,c), which was attributed to the release of OH^−^ by microalgae after absorbing acetate. Similar phenomenon could be found in *Chlorella sorokiniana* [49]. The above results indicated that *Tetraselmis subcordiformis* used acetate as a carbon source, which enhanced cell growth and biomass accumulation.

The addition of 2% CO_2_ as a carbon source (CO_2_-Ac-0) resulted in a significant acceleration of cell growth (OD_750_) compared to that without an additional CO_2_ supply (Air-Ac-0), and the cell dry weight reached a maximum value of 2.27 g/L on day 3, which was 42.8% higher than that under Air-Ac-0 (Figure S1b and Figure 1b,d), indicating that a sufficient CO_2_ supply was crucial for the improvement of cell growth and biomass production in *Tetraselmis subcordiformis*. The addition of 2.5–5 g/L acetate (CO_2_-Ac-2.5 and CO_2_-Ac-5) as an organic carbon source under 2% CO_2_ could effectively promote the cell growth and biomass accumulation (Figure S1b and Figure 1b,d). The addition of 2.5 g/L of acetate (CO_2_-Ac-2.5) led to the most effective promotion of cell growth, with the highest cell dry weight reaching 3.78 g/L within five days, representing a 66.5% increase compared to that without acetate addition (CO_2_-Ac-0). In general, higher biomass yields were obtained with CO_2_-Ac compared to Air-Ac when acetate concentrations were not higher than 5 g/L (Figure 1b). The addition of high concentration of acetate (10 g/L, CO_2_-Ac-10) exerted a strong inhibitory effect on algal cells, with cell growth and biomass production reduced by 38.7% and 44.9%, respectively, in three days compared with the control (CO_2_-Ac-0). Accordingly, biomass productivity was enhanced by 45.0~68.3% in Air-Ac-2.5 and Air-Ac-5 compared to Air-Ac-0, reaching 0.71~0.83 g/L/day on day 3. In contrast, Air-Ac-10 exhibited a significant decline in biomass productivity compared to Air-Ac-0, dropping to only 0.15 g/L/day on day 3 (Figure 1d). Acetate is soluble in cell-membrane lipids, and a high concentration of acetate could affect phosphate membrane transport and inhibit the production of ATP, which interferes with microalgal cell growth [50,51]. In addition, when acetic acid was used as an organic carbon source for microalgae cultivation, succinate could have been excessively produced, which inhibited cell growth [52].

Under CO_2_-Ac conditions, the acetate concentration decreased predominantly on day 1, with the decline rate increasing proportionally to the acetate concentrations, which was similar to that under Air-Ac conditions (Figure 2a,b). The overall acetate decrement in CO_2_-Ac exceeded that in Air-Ac, indicating that the CO_2_ supply could have facilitated acetate uptake. Similar phenomenon has been recorded in *Chlorella sorokiniana* [53]. Therefore, microalgal biomass accumulation could be more effectively promoted with appropriate concentrations of acetate addition (<5 g/L) under CO_2_-enriched conditions. The culture pH remained stable at 5.9–6.4 when 2% CO_2_ served as the carbon source (CO_2_-Ac-0), whereas the pH increased significantly on day 1 with the addition of 2.5–10 g/L acetate and finally stabilized at 7.5–7.7, which was significantly lower than the pH (9.6–9.7) under Air-Ac systems (Figure 2c). This phenomenon likely resulted from the partial neutralization of OH^−^ (released during acetate assimilation) by the higher CO_2_ concentrations and the establishment of a CO_2_-HCO_3_^−^ buffer system that reduced and stabilized the pH, as was also observed previously in *Chlorella vulgaris* [54].

These results demonstrated that *Tetraselmis subcordiformis* can simultaneously utilize both CO_2_ and acetate as carbon sources for cell growth and biomass accumulation, with CO_2_ (as inorganic carbon) and acetate (as organic carbon) exhibiting positive synergistic effects, which was consistent with the mixotrophic cultivation mode using acetate as an organic carbon source previously reported in *Haematococcus pluvialis* [55]. The utilization of acetate in microalgae primarily involves the generation of Acetyl-CoA catalyzed by Acetyl-CoA synthase which requires the participation of ATP [52]. Under sufficient inorganic carbon conditions, microalgal cells maintain a highly efficient photosynthesis, enabling a sufficient ATP production to support acetate uptake and utilization. Acetate can be metabolized through the TCA cycle and glyoxylate cycle to generate additional ATP and intermediate metabolites that provide both energy and carbon skeletons for various physiological metabolic activities in cytoplasm and chloroplasts, which could in return potentially enhance CO_2_ fixation and cell growth [52,55,56]. In addition, it was reported that appropriate acetate concentrations can increase the level of ribulose-1,5-bisphosphate (a key substrate for carbon fixation) and enhance RuBisCO activity [33], thereby promoting photosynthetic carbon fixation and biomass accumulation. Furthermore, CO_2_ supplementation maintained the culture pH at 7.5–7.7 (Figure 2d), which represented the optimal pH range for biomass production in *Tetraselmis subcordiformis* [39] and consequently facilitated cell growth and biomass accumulation. In fact, the combined supply of acetate and CO_2_ enabled an auto-buffering system for the nitrate-deficient cultivation system in which the initial pH stayed relatively low (pH of 5~6), yet no increase occurred with the algal growth (Figure 2c,d, Air-Ac-0 and CO_2_-Ac-0) because of the absence of nitrate assimilation. Meanwhile, the lacked buffering ability of the ASW-N medium caused by the absence of bicarbonate compared with natural seawater (Table S1) could also be compensated for by means of this strategy, which could be of particular interest to industrial cultivations that may reduce the pH-monitoring investment.

Notably, a high concentration of acetate (10 g/L) exhibited strong inhibitory effects on algal growth under 2% CO_2_ conditions (CO_2_-Ac-10), while it showed promotive effects in Air-Ac systems (Air-Ac-10) (Figure 1), suggesting that the threshold for acetate-induced inhibition was significantly lowered under a CO_2_-enriched environment. This may be attributed to the substantially lower culture pH under high CO_2_ conditions (~7.6) compared to the air systems (~9.6). The equilibrium of CH_3_COOH/CH_3_COO^−^ is affected by a low pH and is more inclined to form protonated CH_3_COOH, which dissociates into H^+^ and CH_3_COO^−^ after entering the cell through diffusion; the former disrupts the proton gradient and affects the generation of ATP, while the latter generates osmotic pressure stress and affects the transport of nutrients (such as sugars and phosphates), resulting in the inhibitory effects [30]. Lacroux et al. [57] found that the inhibitory threshold concentration of CH_3_COOH for different algae species varied between 41–207 mg/L. In this study, based on the measured pH and total acetate concentration (C_tot_), the undissociated acetic acid concentration ([CH_3_COOH]) was estimated using the formula [CH_3_COOH] = C_tot_ [H^+^]/(K_a_ + [H^+^]) [30]. The calculations revealed that during the initial cultivation phase, the CO_2_-Ac-10 culture reached a [CH_3_COOH] of 57.22 mg/L, while the Air-Ac-10 culture maintained a [CH_3_COOH] of less than 0.2 mg/L (Table S2), explaining the observed inhibition in CO_2_-Ac-10. In fact, CO_2_-Ac-5 already showed a partial inhibitory effect due to its high level of [CH_3_COOH] (16.89 mg/L, Table S2), which resulted in a lower cell growth and biomass accumulation than that of CO_2_-Ac-2.5 (Figure S1b and Figure 1b,d). In all, the addition of 2.5 g/L of acetate at 2% CO_2_ could provide optimal inorganic and organic carbon sources as well as a favorable environment for *Tetraselmis subcordiformis* to achieve the highest biomass yield.

#### 3.1.2. Photosynthetic Performance

The addition of acetate and CO_2_ significantly impacted the photosynthetic activity of algal cells, consequently affecting biomass accumulation. The *F_v_/F_m_*, representing the maximum quantum yield of photosystem II (PSII), exhibits an inverse correlation with microalgal stress levels [58]. Under N-deficient cultivation, algal cells rely on intracellular protein turnover to sustain growth, particularly through the degradation of photosynthesis-related proteins that cannot be repaired, leading to a photooxidative damage state typically characterized by the decline of *F_v_/F_m_* values [58]. The ABS/RC, indicating the photon flux absorbed per active reaction center, reflects the antenna size and serves as another important stress indicator [58]. Under N-deficient conditions, the reduction in active reaction centers causes antenna proteins to be redistributed to the remaining centers, resulting in increased ABS/RC values [58,59].

**Figure 3:**
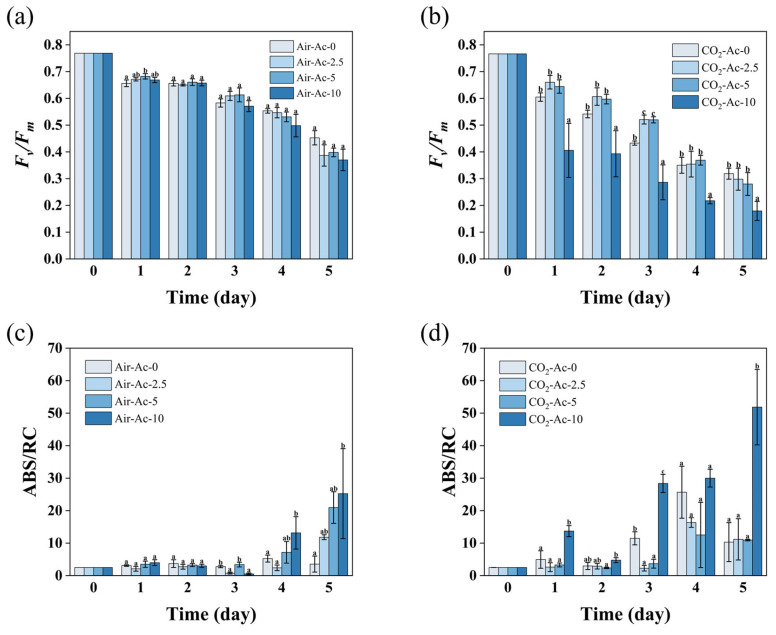
The photosynthetic performance indicated by *F_v_/F_m_* (**a**,**b**) and ABS/RC (**c**,**d**) in *Tetraselmis subcordiformis* under different concentrations of acetate (0 g/L, 2.5 g/L, 5 g/L, and 10 g/L) conditions with air (**a**,**c**) or 2% CO_2_ (**b**,**d**) aerations during the N-deficient cultivation process. The different letters above the column in the same cultivation day represent the significant difference (*p* < 0.05) among various cultivation conditions.

As shown in Figure 3a, in the absence of additional carbon sources (Air-Ac-0), *F_v_/F_m_* gradually decreased from an initial level of 0.77 to 0.45 over five days due to N-deficient stress. Acetate supplementation (2.5 g/L–10 g/L, Air-Ac-2.5–Air-Ac-10) showed negligible effects on photosynthetic efficiency during the first three days, but by day 5, *F_v_/F_m_* declined to 0.35–0.40, representing a 12.1–22.5% reduction compared to the control (Air-Ac-0). Accordingly, ABS/RC in Air-Ac-0 showed a gradual increase, reaching 3.55 by day 5. While ABS/RC in the acetate-supplemented cultures (Air-Ac-2.5–Air-Ac-10) initially resembled the control, it exhibited a remarkable elevation by day 5, reaching levels of 11.83–30.66, which represented a 2–7-fold increase over the control (Air-Ac-0, *p* < 0.05). These results demonstrate that under inorganic carbon limitation, acetate, as an organic carbon source, exacerbated photooxidative stress when the microalgae were exposed to N deficiency. This may stem from the fact that acetate enabled the partial relief of carbon limitation, which increased the C/N ratio and consequently accelerated cell growth (as discussed in Section 3.1.1) and non-nitrogenous biomass accumulation (e.g., starch, as shown in Section 3.1.3). Consequently, the intracellular protein turnover was promoted, ultimately intensifying photooxidative damage effects. As could be seen in Figure S2, the intracellular protein content after adding acetate (Air-Ac-2.5–Air-Ac-10) was significantly reduced by 24.3–38.0% compared with the control (Air-Ac-0), which supported the rationality of the above inference.

**Figure 4:**
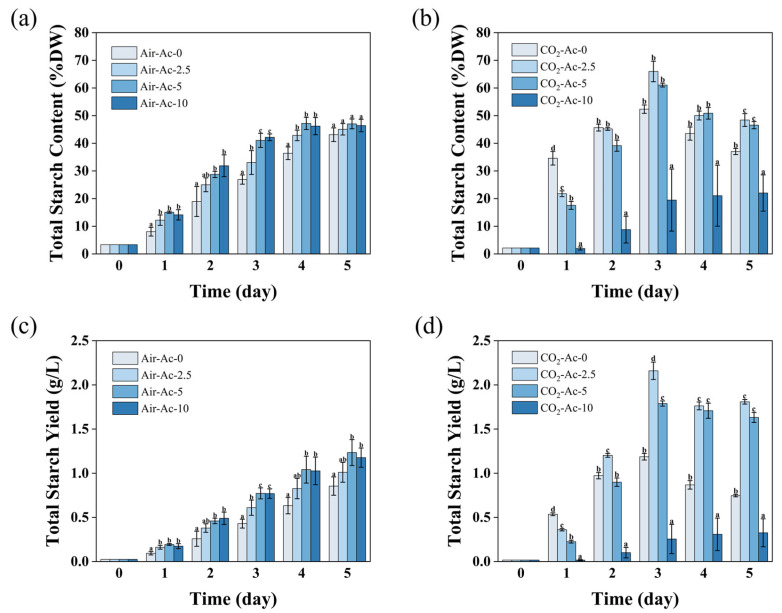
The total starch content (**a**,**b**) and total starch yield (**c**,**d**) in *Tetraselmis subcordiformis* under different concentrations of acetate (0 g/L, 2.5 g/L, 5 g/L, and 10 g/L) conditions with air (**a**,**c**) or 2% CO_2_ (**b**,**d**) aerations during the N-deficient cultivation process. The different letters above the column in the same cultivation day represent the significant difference (*p* < 0.05) among various cultivation conditions.

When 2% CO_2_ was supplied (CO_2_-Ac-0), the decline in photosynthetic efficiency occurred more rapidly than in Air-Ac-0, with *F_v_/F_m_* decreasing to 0.32 by day 5, which was 29.6% lower than Air-Ac-0. The ABS/RC showed a rapid elevation, peaking at 25.67 on day 4 and was 3.02-fold higher than in Air-Ac-0. This accelerated photoinhibition could result from the CO_2_-induced elevation of the C/N ratio, which enhanced both the cell growth (Figure S1b) and starch accumulation (Figure 4b) while promoting intracellular protein turnover (evidenced by the 16.3% lower protein content in CO_2_-Ac-0 versus Air-Ac-0, Figure S2), ultimately exacerbating photooxidative damage effects, similar to that of acetate supplementation in Air-Ac-0. In contrast to Air-Ac conditions, the acetate addition under the 2% CO_2_ supply (2.5 g/L and 5 g/L, CO_2_-Ac-2.5 and CO_2_-Ac-5) was able to alleviate the photooxidative damage on algal cells produced by N deficiency, especially in the first three days when *F_v_/F_m_* was maintained at around 0.52, a level representing 20% of the enhancement compared with that in the control group (CO_2_-Ac-0) (Figure 3b, *p* < 0.05). Accordingly, ABS/RC in CO_2_-Ac-2.5 and CO_2_-Ac-5 decreased markedly by 68.0–80.1% relative to CO_2_-Ac-0 (Figure 3d, *p* < 0.05). Previous studies demonstrated that mixotrophic cultivation with an organic carbon supply could alleviate photooxidative damage and enhance stress tolerance in microalgae [60], thereby benefiting biomass accumulation, which was consistent with our findings herein. Acetate supplementation may trigger adaptive responses including photosynthetic pigment remodeling (reduced chlorophyll synthesis with a concomitant enhancement of xanthophyll production) and the activation of non-photochemical quenching (NPQ) mechanisms, collectively reducing photodamage and protecting cells against oxidative stress [61]. The supply of acetate could upregulate the expression of PS I apparatus (e.g., psaE), which contributes to the cyclic electron flow and facilitates the NPQ function under oxidative stress (e.g., high light stress under N deficiency) [62,63]. Furthermore, the increased CO_2_ fixation efficiency caused by the addition of acetate [33] could accelerate the consumption of ATP and NADPH, and thus may exert a photoprotective role by providing sufficient electron acceptors for PSI and avoiding the formation of an excessive reduction state of electrons in the thylakoid membrane [64]. The addition of a high acetate concentration (10 g/L, CO_2_-Ac-10) imposed severe stress on algal cells, causing *F_v_/F_m_* to sharply decline to 0.18 within five days, which showed a 44.2% reduction compared to the control group (CO_2_-Ac-0, Figure 3b). Concurrently, ABS/RC significantly increased to 60.08, representing a 4-fold elevation over the control (CO_2_-Ac-0, Figure 3b,d). This excessive acetate likely elevated intracellular reactive oxygen species (ROS) levels [34], disrupting the PSII complex and thus significantly decreasing photosynthetic efficiency [65]. The decline in photosynthetic activity under the excessive acetate supply strongly inhibited both cell growth and biomass accumulation (as discussed in Section 3.1.1).

These results indicated that acetate could promote algal cell growth and biomass accumulation through potentially different mechanisms under inorganic carbon-deficient (Air-Ac) and sufficient (CO_2_-Ac) conditions. In Air-Ac, acetate mainly served as an organic carbon and energy source, while in CO_2_-Ac, it may additionally have alleviated oxidative stress to enhance photosynthetic efficiency, and ultimately facilitated biomass production.

#### 3.1.3. Starch Production

**Figure 5:**
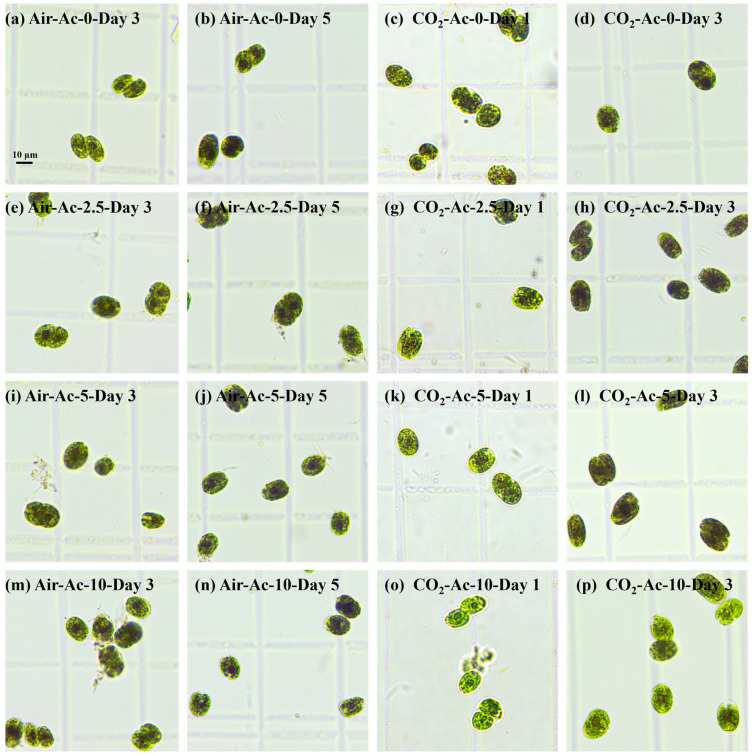
Starch accumulation revealed by iodine staining in *Tetraselmis subcordiformis* under different concentrations of acetate (0 g/L, 2.5 g/L, 5 g/L, and 10 g/L) conditions with air (**a**,**b**,**e**,**f**,**i**,**j**,**m**,**n**) or 2% CO_2_ (**c**,**d**,**g**,**h**,**k**,**l**,**o**,**p**) aerations during the N-deficient cultivation process.

Under nitrogen-deficient (-N) conditions, *Tetraselmis subcordiformis* primarily directs photosynthetically fixed carbon toward starch synthesis [21,66]. Acetate supplementation significantly affects starch production in this alga under N-deficient conditions. As shown in Figure 5 and Table 2, without additional carbon sources (Air-Ac-0), the microalgae slowly synthesized starch using limited atmospheric CO_2_, and the starch content, yield, and productivity reached 43.1%, 0.85 g/L, and 0.17 g/L/day by day 5, respectively. The addition of acetate accelerated the production of starch by the microalgae. During the early cultivation stage (days 1–4) with 2.5–10 g/L of the acetate supply under air conditions (Air-Ac-2.5~Air-Ac-10), both the starch content and yield showed significant increases compared to the control (Air-Ac-0) (*p* < 0.05, Figure 4a,c). Particularly in Air-Ac-5 and Air-Ac-10, starch accumulation accelerated markedly, with the starch content reaching maximum values of 47.2% by day 4, which was 29.7% higher than that of the control (Air-Ac-0). The starch content in Air-Ac-5 and Air-Ac-10 was still slightly higher than that of the control (Air-Ac-0) on day 5, while starch yields (1.18–1.23 g/L) were significantly greater (37.7–44.4% improvement, *p* < 0.05, Figure 4c). The iodine staining of algal cells (Figure 5a,i,m) revealed darker cells in Air-Ac-5 and Air-Ac-10 compared to Air-Ac-0 on day 3, further confirming that acetate supplementation accelerated intracellular starch accumulation in *Tetraselmis subcordiformis*. It is generally believed that after the acetate is converted into acetyl-CoA in cells, it enters the glyoxylic acid cycle and generates succinate, which can be further converted into oxaloacetate [30]. In the presence of ATP, oxaloacetate can be converted into phosphoenolpyruvate (PEP) under the catalysis of PEP carboxykinase, which triggers gluconeogenesis to synthesize starch [67]. Therefore, under inorganic carbon-limited conditions, acetate can serve as a carbon source to promote starch synthesis in microalgae. Similar phenomena have also been observed in cultures of *Chlamydomonas reinhardtii* and *Chlorella pyrenoidosa* [63,68].

Under 2% CO_2_ as an inorganic carbon source (CO_2_-Ac-0), the starch accumulation was significantly accelerated compared with that under Air-Ac-0, with both the starch content and yield being markedly higher than the levels under Air-Ac-0 (Figure 4a–d). In CO_2_-Ac-0, starch accumulation reached its maximum value on day 3, with the starch content, yield, and productivity reaching 52.4%, 1.19 g/L, and 0.39 g/L/day, respectively, representing 21.4%, 38.9%, and 134.9% increases compared to Air-Ac-0 (Figure 4a–d, Table 2). The observation of starch accumulation in cells by iodine staining on day 3 was consistent with the above results (Figure 5a,d). The addition of CO_2_ supplemented the inorganic carbon source, increased the cellular C/N ratio, and promoted photosynthetic starch synthesis, which was consistent with the results in *Chlorella sorokiniana* [23].

Unlike under Air-Ac-0 conditions, acetate addition under 2% CO_2_ conditions showed an obvious inhibition on starch accumulation in the early stage, although a significant promotion could be observed in the later stage. As shown in Figure 4b,d, under 2% CO_2_ conditions, the addition of 2.5–5 g/L acetate (CO_2_-Ac-2.5 and CO_2_-Ac-5) showed a significantly lower starch content (17.6% vs. 21.8%) and yield (0.23 g/L vs. 0.36 g/L) than the control group CO_2_-Ac-0 on day 1 (*p* < 0.05); subsequently, starch accumulation gradually accelerated and exceeded the control group by day 3. The observation of starch accumulation in cells on days 1 and 3 by iodine staining was also consistent with the above results (Figure 5g–i,l). The inhibitory effect on starch accumulation in the early stage after acetate addition under 2% CO_2_ conditions may be due to the photoprotective effect of acetate (as discussed in Section 3.1.2), which reduced the stress level imposed on the algal cells. Stress is usually a prerequisite for starch accumulation in microalgae [13]. In fact, it could also be seen from the changes in protein content that on the first day of cultivation, the protein content of the cells in CO_2_-Ac-2.5 and CO_2_-Ac-5 was significantly higher than that of CO_2_-Ac-0 (Figure S2), indicating that the protein turnover and the consequent stress were both lower. On the 3rd and 5th days, the protein content of the cells in CO_2_-Ac-2.5 and CO_2_-Ac-5 was gradually reduced relative to that of CO_2_-Ac-0 (Figure S2), indicating that the protein turnover in cells increased with the elevation of stress levels, and therefore the starch accumulation ability was significantly improved. The addition of 10 g/L acetate (CO_2_-Ac-10) strongly inhibited starch accumulation, with both the starch content and yield being significantly lower than the control group (Figure 4b,d and Figure 5o,p, *p* < 0.05). This was attributed to the inhibition of photosynthesis by high concentrations of acetate (Figure 3b,d), which imposed excessive stress on algal cells and greatly affected cell growth and biomass production.

Collectively, this study demonstrated that adding 2.5 g/L of acetate under 2% CO_2_ (CO_2_-Ac-2.5) was the best carbon source supply strategy for promoting starch accumulation in *Tetraselmis subcordiformis* (Figure 4b,d), which coincided with the optimal conditions for biomass production mentioned earlier (Figure 1b,d). The maximum starch content and yield under this condition reached 66.0% and 2.16 g/L, respectively, and the maximum starch productivity reached 0.71 g/L/day, which were 26.0%, 82.0%, and 37.8% higher than the control (CO_2_-Ac-0), respectively. It was noteworthy that the addition of acetate under the 2% CO_2_ supply promoted microalgal starch production more significantly compared with that under air, with a substantial increase in starch content and yield (16.6–26.0%/50.8–82.0% in CO_2_ vs. 4.5–9.0%/18.2–44.4% in air). The reason for this may be that the addition of acetate at 2% CO_2_ partially mitigated the stress suffered by the cells in the early stage of cultivation, resulting in the maintenance of a higher photosynthetic efficiency (as described in Section 3.1.2), which was more favorable for starch production in the later stage. In fact, although stress is required to induce microalgal starch accumulation, maintaining a sufficient photosynthetic efficiency is crucial for efficient starch production since starch synthesis requires carbon sources and energy [13]. Additionally, the combination of 2% CO_2_ and an appropriate acetate supply established an effective pH buffer system (as discussed in Section 3.1.1), ensuring cells with a favorable pH (7.5–7.7) for starch accumulation [69], which thereby significantly enhanced the starch production capacity.

Interestingly, this study also found that the addition of acetate had little effect on lipid accumulation, which was different from the phenomenon reported in most of the literature that acetate promoted lipid accumulation in microalgae (such as *Chlorella vulgaris*, *Chlamydomonas reinhardtii*, and *Dunaliella salina*) [35,70,71]. As a starch-producing microalga, *Tetraselmis subcordiformis* may primarily metabolize acetate through the glyoxylate cycle, generating oxaloacetate that enters gluconeogenesis to produce starch. The specific mechanisms require further investigation. Nevertheless, this study further highlighted the high efficiency of *Tetraselmis subcordiformis* in directing carbon sources towards starch biosynthesis rather than lipids, which was especially favorable for exclusive starch production.

The composition of amylose (Am) and amylopectin (Ap) in starch significantly influences its properties and downstream applications [22]. A further analysis of the starch composition revealed that the starch of *T. subcordiformis* cultivated under the condition of air (Air-Ac-0) had an Am/Ap of 0.49 on day 5, whereas the addition of 2.5–10 g/L of acetate (Air-Ac-2.5–Air-Ac-10) increased the Am/Ap to 0.56–0.64 (Table 2), indicating that acetate promoted Am more effectively than Ap. The Am/Ap of the starch obtained from the cultivation of *T subcordiformis* under 2% CO_2_ (CO_2_-Ac-0) reached 0.55–0.81, which was generally higher than that of Air-Ac-0 (0.49–0.55), indicating that an adequate CO_2_ supply was more favorable for Am synthesis. These results are inconsistent with the reports in the literature that the Am/Ap ratio of the starch in *Chlorella kessleri* and *Chlamydomonas reinhardtii* was higher at a low CO_2_ (air) than at a high CO_2_ (2–3%) [23,29]. In those studies, a low CO_2_ was found to induce the expression of granule-bound starch synthase (GBSS), the key enzyme for amylose synthesis, and was also closely related to the CO_2_ concentrating mechanism (CCM), with the starch mainly accumulated around pyrenoid (pyrenoid starch) [29,72]. In this study, starch synthesis was induced under nitrogen-deficient (-N) conditions, with starch primarily synthesized in chloroplast stroma (stroma starch, Figure 5), which differed from the formation of pyrenoid starch under N-replete conditions in the above studies, and thus there may be a difference in the process of the formation of the two types of starch in response to the CO_2_ concentration.

Unlike under air, the Am/Ap of microalgal starch obtained by adding 2.5–5 g/L of acetate (CO_2_-Ac-2.5 and CO_2_-Ac-5) under 2% CO_2_ was gradually decreased (Table 2). Particularly on day 3 when starch accumulation peaked, Am/Ap significantly decreased from 0.81 to 0.73 (*p* < 0.05), indicating that acetate promoted Ap synthesis more effectively than Am under these conditions. A high acetate concentration (CO_2_-Ac-10) inhibited starch accumulation and substantially reduced Am/Ap to 0.31–0.45 (Table 2). In all, CO_2_ and acetate additions not only significantly improved the starch yield in *T. subcordiformis*, but also changed the starch composition (Am/Ap).

#### 3.1.4. Enzyme Activity Related to Starch Metabolism

The starch yield and composition are closely related to the activity of the enzymes involved in its synthetic enzymes. ADP-glucose pyrophosphorylase (AGPase), which effectively catalyzes the formation of ADP-glucose from Glucose-1-phosphate, is considered as the committed step in starch biosynthesis [73], while branching enzyme (BE) and isoamylase (ISA), which perform starch branching and debranching functions, respectively, are the key enzymes determining the amylose/amylopectin composition [10]. Therefore, in this study, the activities of AGPase, BE, and ISA were determined under optimal conditions of starch accumulation in *T. subcordiformis* at different CO_2_ concentrations and acetate additions (Air-Ac-0 vs. Air-Ac-5–5 day and CO_2_-Ac-0 vs. CO_2_-Ac-2.5–3 day).

**Figure 6:**
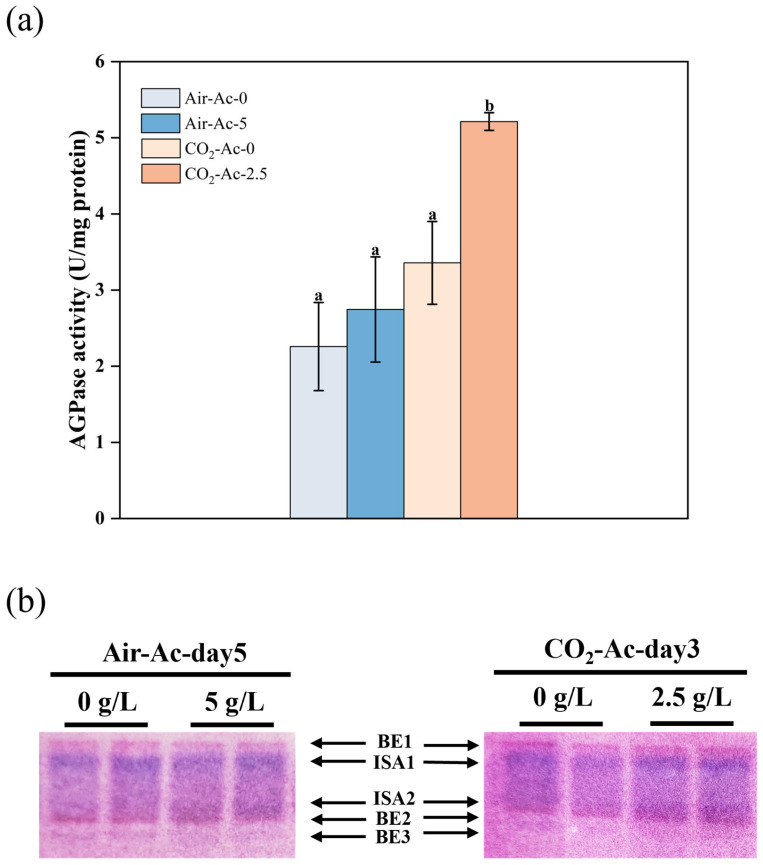
The activity of AGPase (**a**) and isoamylase and the branching enzyme (**b**) in *Tetraselmis subcordiformis* under different concentrations of acetate with air (day 5, 0, and 5 g/L of acetate) or 2% CO_2_ (day 3, 0, and 2.5 g/L of acetate) aerations during the N-deficient cultivation process. The different letters above the column for AGPase (**a**) represent the significant difference (*p* < 0.05) among various cultivation conditions.

As shown in Figure 6a, the AGPase activity exhibited the lowest level (2.26 U/mg pro) under air (Air-Ac-0). With 5 g/L of acetate addition (Air-Ac-5), the AGPase activity slightly increased to 2.75 U/mg pro, representing a 21.5% improvement over the Air-Ac-0, which was in agreement with the starch accumulation trend (Figure 4a,c). The AGPase activity further increased to 3.36 U/mg pro under the addition of 2% CO_2_ as an inorganic carbon source (CO_2_-Ac-0), with the value being 48.7% higher than that of Air-Ac-0, which explained the higher production under this condition relative to that of Air-Ac-0. The addition of 2.5 g/L of acetate (CO_2_-Ac-2.5) at 2% CO_2_ dramatically increased the AGPase activity to 5.21 U/mg pro (Figure 6a, *p* < 0.05), representing a 55.2% enhancement compared to that at CO_2_-Ac-0, which coincided with the highest starch accumulation under this condition (Figure 4b,d). In *Chlorella pyrenoidosa*, the addition of acetate induced the up-regulation of AGPase, thereby enhancing starch synthesis [63], which was consistent with the findings of the present study. These results demonstrated that acetate addition under a sufficient inorganic carbon supply could effectively enhance AGPase activity, thereby promoting starch production in *Tetraselmis subcordiformis*.

The zymogram-based assay of ISA and BE activities revealed that the BE3 activity of CO_2_-Ac-0 was significantly lower than that of Air-Ac-0, whereas there was no significant difference in the activity of ISA (Figure 6b), suggesting that the addition of CO_2_ reduced the branching activity of the starch, resulting in a decrease in the relative proportion of Ap and an increase in the ratio of Am (elevated Am/Ap, Table 2). This could be similar to the case in the production of high-amylose starch found in crops where the inhibition of starch branching enzymes (BEs) decreases the degree of branching and produces “Am-like” material in Ap biosynthesis [10,74]. In the Air-Ac cultivation system, the addition of 5 g/L of acetate slightly promoted ISA2 and significantly inhibited BE3 (Figure 6b), and the overall ISA/BE ratio increased, indicating that the addition of acetate promoted the debranching of starch while inhibiting its branching, which therefore led to a significant increase in Am/Ap (Table 2). In *Chlorella pyrenoidosa*, the supply of acetate under air resulted in an increase of BE transcription, which was different from the case here in *Tetraselmis subcordiformis* [63]. In high-amylose maize starch, a high expression of the isoamylase 2 (ISA2) with a simultaneously low expression of the starch branching enzyme IIb (SBEIIb) enabled the increase of the relative amylose content due to the deficient branching and excessive debranching process, which could also be applied herein [75]. On the contrary, in the CO_2_-Ac cultivation system, the addition of 2.5 g/L of acetate significantly increased the activity of BE2 (Figure 6b), leading to a significant increase in the Ap ratio and a decrease in Am/Ap (Table 2). In conclusion, the addition of CO_2_ and acetate altered the activities of enzymes related to starch synthesis in *T. subcordiformis*, causing significant changes in starch yield and composition.

### 3.2. The Characterization of Starch Produced Under Different Carbon Sources

Starch was extracted from *T. subcordiformis* cultivated under six typical carbon source conditions (Air-Ac-0, Air-Ac-5, Air-Ac-10, CO_2_-Ac-0, CO_2_-Ac-2.5, and CO_2_-Ac-5) and characterized in terms of granule morphology, ordered structure, and in vitro digestibility. As shown in Table 3, the Am/Ap ratio of the extracted starch showed minimal deviation with consistent variation trends across all conditions compared with the results of the in vivo assay, indicating a successful starch extraction.

#### 3.2.1. Morphology

**Figure 7:**
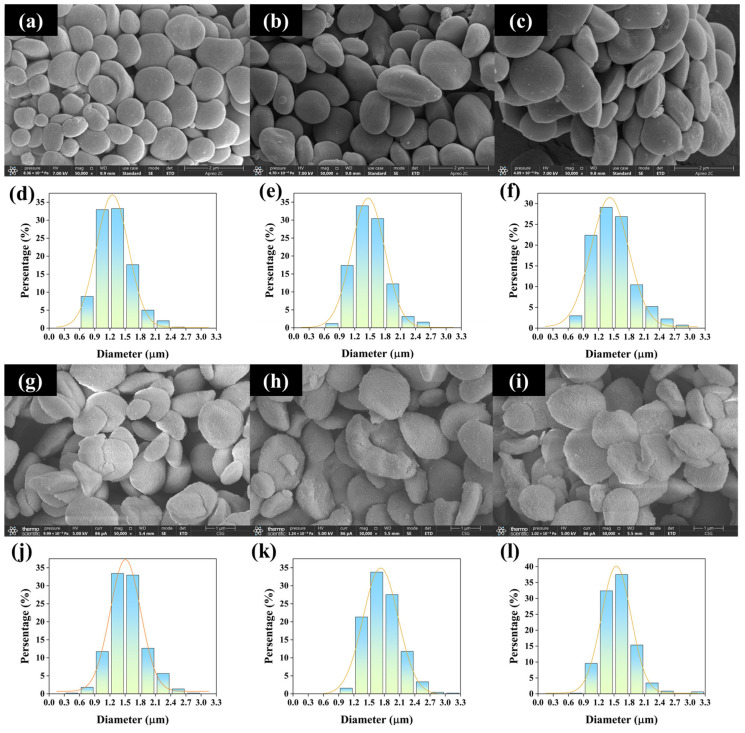
A morphology analysis (**a**–**c**,**g**–**i**) measured by SEM and the size distribution (**d**–**f**,**j**–**l**) of starch from *Tetraselmis subcordiformis* cultivated under different concentrations of acetate (0 g/L, 2.5 g/L, 5 g/L, and 10 g/L) conditions with air (Air-Ac, 5 days, (**a**–**f**)) or 2% CO_2_ (CO_2_-Ac, 3 days, (**g**–**l**)) aerations during the N-deficient cultivation process ((**a**,**d**): Air-Ac-0; (**b**,**e**): Air-Ac-5; (**c**,**f**): Air-Ac-10; (**g**,**j**): CO_2_-Ac-0; (**h**,**k**): CO_2_-Ac-2.5; (**i**,**l**): CO_2_-Ac-5).

The morphology of microalgal starch granules was analyzed using scanning electron microscopy. As shown in Figure 7, most of the *T. subcordiformis* starch granules exhibited a discoidal shape, which was similar to the starch morphology of other microalgae such as *Chlorella* sp. and *Chlamydomonas reinhardtii* [22,26,47]. The granule size of *T. subcordiformis* starch predominantly ranged from 0.9 to 2.1 μm (Figure 7), which was larger than that of *Chlorella* sp. and *Chlamydomonas reinhardtii* [26,76]. When cultivated without additional carbon sources under air conditions (Air-Ac-0), *T. subcordiformis* produced starch granules with an average size of 1.30 μm (Figure 7d, Table 3). Supplementation with 5–10 g/L of acetate (Air-Ac-5 and Air-Ac-10) resulted in plumper and thicker starch granules with average sizes increasing to 1.51–1.55 μm (*p* < 0.05, Figure 7e,f and Table 3). Under CO_2_-enriched conditions (CO_2_-Ac), the starch granules appeared flatter yet generally larger than those from Air-Ac cultures (Figure 7j–l), with rougher surfaces and more irregular shapes. Typically, starch granules from CO_2_-Ac-0 showed an average size of 1.54 μm, representing an 18.5% increase over Air-Ac-0 (*p* < 0.05, Table 3). This was in contrast to the observations in *Chlorella kessleri* and *Chlorella sorokiniana* that CO_2_ generally induced smaller starch granules [23,29]. With the 2.5 g/L acetate addition under the CO_2_ supply (CO_2_-Ac-2.5), the average granule size increased to 1.76 μm (14.3% larger than CO_2_-Ac-0, Figure 7j,k, Table 3). No significant difference in size was observed between CO_2_-Ac-5 and CO_2_-Ac-0 (Figure 7j,l and Table 3). Overall, both CO_2_ and acetate supplementation altered the morphology of *T. subcordiformis* starch granules, suggesting potential structural changes as well.

#### 3.2.2. Ordered Structure

**Figure 8:**
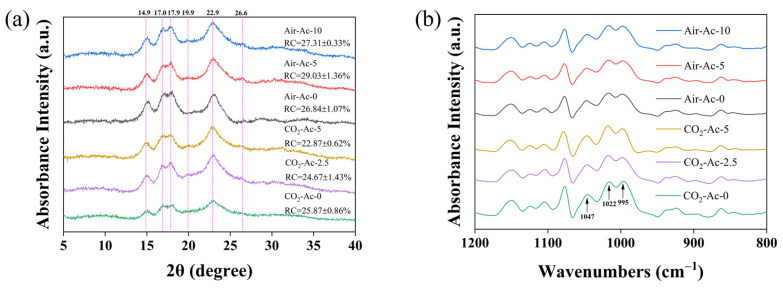
XRD (**a**) and deconvoluted FTIR spectra (**b**) in *Tetraselmis subcordiformis* under different concentrations of acetate (0 g/L, 2.5 g/L, 5 g/L, and 10 g/L) conditions with air (Air-Ac, 5 days) or 2% CO_2_ (CO_2_-Ac, 3 days) aerations during the N-deficient cultivation process.

Starch has a long-range ordered structure, which can be evaluated by X-ray diffraction (XRD) patterns [77]. As shown in Figure 8a, the XRD spectra of microalgal starch harvested under all six conditions showed characteristic peaks at 14.9°, 16.9°, 17.9°, and 22.9° (2θ), corresponding to typical A-type crystalline structures [77]. This observation aligned with the structural characteristics previously reported for *Tetraselmis subcordiformis* starch [22,78]. CO_2_ supplementation did not alter the crystalline type of *T*. *subcordiformis* starch. Similar findings were reported for *Chlorella* and *C. reinhardtii*, where variations in the CO_2_ concentration also preserved the A-type crystalline structure [29,76]. The relative crystallinity (RC) of *T*. *subcordiformis* starch under both CO_2_ and acetate conditions ranged from 22% to 30%, which was typical for common microalgal starch [22,26,47]. However, the RC of *T*. *subcordiformis* starch obtained under air (Air-Ac) was generally higher than that of the starch under CO_2_ (CO_2_-Ac) (27.3–29.0% vs. 22.9–25.9%, Table 3), which suggested an enhanced long-range structural order in the starch from the Air-Ac cultivation conditions. This may be because the starch under this condition contained a lower proportion of Am (0.59–0.70 vs. 0.62–0.75, Table 3), and Am is generally considered to participate in the formation of amorphous regions of starch, which is negatively correlated with RC [79]. Additionally, an increased amylose content may disrupt the ordered packing of amylopectin double helices in crystalline lamellae [80], resulting in a decrease in crystallinity. Acetate addition showed no significant effect on RC under either air or CO_2_ conditions (*p* > 0.05). Intriguingly, acetate supplementation showed two additional weak peaks at 19.9° and 26.6° (2θ) (Figure 8a). The 19.9° (2θ) peak represents V-type starch crystals composed of starch–lipid complexes [81], suggesting that acetate was able to induce the formation of trace amounts of amylose–lipid complexes in *T*. *subcordiformis*. In the B-type amylose crystal structure [82], 26.6° (2θ) is generally found, suggesting that acetate addition may induce new crystalline forms in the starch of *T*. *subcordiformis*.

FTIR spectra can effectively evaluate the short-range ordered structure of starch [83]. As shown in Figure S3, the FTIR spectra of *T. subcordiformis* starch cultivated under different carbon sources showed no significant differences in the ranges of 3000–3700 cm^−1^ (-OH stretching vibration), 2900–3000 cm^−1^ (C-H stretching vibration), 1600–1700 cm^−1^ (H-O-H bending vibration), 1120–1180 cm^−1^ (C-O, C-C, C-O-H stretching vibrations), and 900–1100 cm^−1^ (C-O-H bending and stretching vibrations) [22], which indicated that the functional groups of the starch in the microalgae cultivated with different carbon sources were not changed. In the IR spectra, 1047 cm^−1^ and 1022 cm^−1^ correspond to crystalline structures of starch aggregates and irregular agglomerations of starch macromolecules, respectively. The R_1047/1022_ ratio from deconvoluted FTIR spectra can be used to quantitatively assess the short-range ordering of starch [83]. As shown in Figure 8b and Table 3, the short-range ordered structure (R_1047/1022_) of starch in *T. subcordiformis* under CO_2_ was generally lower than that from air cultivation (1.21–1.26 vs. 1.24–1.31), while acetate addition showed no significant effect on R_1047/1022_ (*p* > 0.05), which was in agreement with the RC results. In summary, both the long- and short-range ordered structure in *T. subcordiformis* starch decreased under inorganic carbon (CO_2_) supply, while organic carbon (acetate) may have contributed to the formation of new starch crystalline forms.

#### 3.2.3. Digestibility

**Figure 9:**
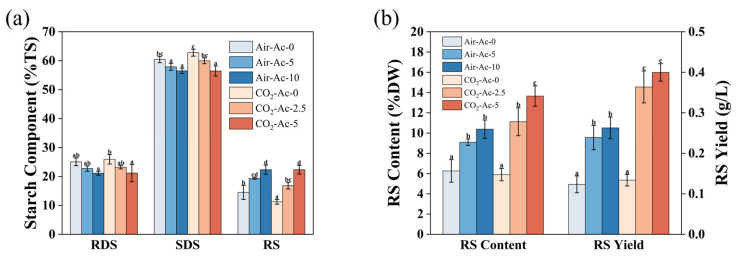
The starch component (%TS, (**a**)), RS content (%DW), and yield (**b**) in *Tetraselmis subcordiformis* under different concentrations of acetate supply with air (Air-Ac, 0 g/L, 5 g/L, and 10 g/L of acetate) or 2% CO_2_ (CO_2_-Ac, 0 g/L, 2.5 g/L, and 5 g/L of acetate) aerations during the N-deficient cultivation process. The different letters above the column for the same parameter represent the significant difference (*p* < 0.05) among various cultivation conditions.

The digestibility of starch determines its downstream applications. Starch can be categorized into rapidly digestible starch (RDS), slowly digestible starch (SDS), and resistant starch (RS) according to its in vitro digestibility [22]. In this study, the in vitro digestibility of starch from *T*. *subcordiformis* under six typical carbon source conditions (Air-Ac-0, Air-Ac-5, Air-Ac-10, CO_2_-Ac-0, CO_2_-Ac-2.5, and CO_2_-Ac-5) was evaluated using the method described by Englyst [48]. As shown in Figure 9a, the RDS, SDS, and RS of the starch from *T*. *subcordiformis* cultured under air (Air-Ac-0) accounted for 25.1%, 60.4%, and 14.5% of the total starch, respectively. Acetate addition in this system reduced the proportions of RDS and SDS while significantly increasing the RS content (*p* < 0.05) in a concentration-dependent manner. Under Air-Ac-5 and Air-Ac-10, the RS content increased to 19.4–22.4%, representing a 33.3–54.0% improvement over the control (Air-Ac-0). Starch from the CO_2_-enriched cultivation (CO_2_-Ac-0) contained 26.0% RDS, 62.8% SDS, and 11.3% RS, with the RS content being 22.5% lower than that under Air-Ac-0 (*p* < 0.05), indicating CO_2_ aeration enhanced the digestibility of the starch in *T. subcordiformis*. Similar results were reported in *Chlorella sorokiniana* [23], that starches from the microalgae cultivated under CO_2_-enriched conditions were hydrolyzed more easily. In the CO_2_-enriched cultures, the starch granules were flatter and the surface was rougher (Figure 7g), which may provide more hydrolysis sites for starch hydrolase, making it more prone to hydrolyzation [22].

Similar to Air-Ac conditions, acetate addition (2.5–5 g/L) under the CO_2_ supply (CO_2_-Ac-2.5 and CO_2_-Ac-5) produced starches with a higher RS content (16.8–22.9%, *p* < 0.05) than CO_2_-Ac-0 (Figure 9a). These results demonstrated that the addition of acetate could significantly reduce the in vitro digestibility of starch in *T*. *subcordiformis*. This may result from the formation of trace amounts of starch–lipid complexes (V-type crystal structure, Figure 8a) under acetate induction, which was conductive to the improvement of the enzymatic hydrolysis resistance of the starch [84]. B-type starch double helices packed in hexagonal unit cells contain 36 water molecules with more intermolecular hydrogen bonds, exhibiting more complex and stable arrangements that are harder to hydrolyze than A-type starch [85,86]. The reduced digestibility of the starch under the addition of acetate may also be partially ascribed to the formation of this B-type crystalline structure, as discussed above (Figure 8a). It should be noted that the Am/Ap ratio exhibited no obvious correlation with the digestibility (i.e., the RS content). This was inconsistent with the general belief that the starch digestion rate negatively correlates with the relative Am content because Am is more easily retrograded which reduces the enzyme susceptibility [87]. In fact, recently, it was found that a rice with a lower amylose content had a higher RS content, probably due to the elongated linearized amylopectin that led to the formation of compact crystallization, making them resistant to hydrolysis [88]. Nevertheless, it implied that other factors (such as the crystalline structure and granule morphology, as discussed above, and fine structures of Am and Ap) rather than Am/Ap determined the digestibility of the starch obtained from *T. subcordiformis*, which deserves further investigation.

The RS content in the cell dry weight (RS%DW) and yield (g/L) are important indicators for evaluating the RS production efficiency. As shown in Figure 9b, the RS content of *T. subcordiformis* cultured without adding any carbon source (Air-Ac-0) was 6.3%DW, and the RS yield reached 0.12 g/L. The addition of acetate in this system significantly increased the RS content and yield (*p* < 0.05) in a concentration-dependent manner. The RS content under Air-Ac-5 and Air-Ac-10 increased to 9.1–10.4%DW, which represented an increase of 45.4–65.8% compared with the control (Air-Ac-0); the yield increased to 0.24–0.26 g/L, demonstrating a 93.8–113.1% enhancement compared with the control (Air-Ac-0). For *T. subcordiformis* cultured under CO_2_-rich conditions (CO_2_-Ac-0), the RS content was 5.9%DW and the yield was 0.13 g/L, which was not significantly different from Air-Ac-0 (Figure 9b, *p* > 0.05). When 2.5–5 g/L of acetate (CO_2_-Ac-2.5 and CO_2_-Ac-5) was added under the 2% CO_2_ supply, the RS content reached 11.1–13.7%DW and the RS yield reached 0.36–0.40 g/L, which were 88.5–131.4% and 172.0–198.8% (*p* < 0.05) higher than those under CO_2_-Ac-0, respectively, with the increase exhibiting a concentration-dependent manner. The above results demonstrated that the addition of acetate can significantly increase the RS content (%DW) and yield in *T. subcordiformis*. This was because the appropriate concentration of acetate can promote the accumulation of biomass (Figure 1) and starch (Figure 4a,b) in microalgal cells, and at the same time promote the RS content (%TS) (Figure 9a), which ultimately led to the achievement of a larger RS content (%DW) and yield.

Overall, the combined application of CO_2_ and acetate as a carbon source can enable *T. subcordiformis* to achieve an optimal RS production efficiency. The maximum RS content based on cell dry weight (13.7%DW) obtained by adding 5 g/L of acetate under 2% CO_2_ (CO_2_-Ac-5) was the highest value of RS%DW reported in microalgae so far (Table 4); the RS content based on the total starch (22.4%TS) and yield (0.40 g/L) obtained under this condition was also significantly higher than that reported in *Chlorella* (a content of 5.2–16.4%TS, and a yield of 0.0025–0.0058 g/L, [25,26,27]), highlighting the advantage of *T. subcordiformis* in producing high value-added functional starch (RS) under this carbon source supply strategy. This study demonstrated that the addition of acetate reduced the proportion of SDS and RDS of microalgae starch and increased the proportion of RS, which could enable the development of low-GI foods from the starch produced by *T. subcordiformis*. Meanwhile, the enhanced RS content should facilitate the production of functional foods used for the management of diabetes and the improvement of intestinal health [7].

### 3.3. A Discussion on the Acetate-CO_2_ Strategy

The present study demonstrated the positive effects of the combined acetate-CO_2_ supply on the functional starch (RS) production from the marine microalga *T. subcordiformis* (Figure 9). The most prominent advantage of the RS production from microalgae over the traditional crops should be the stability of product quality and productivity due to the controllable cultivation conditions in bioreactors instead of the cultivation in fields. The theoretically much higher productivity of microalgal starch relative to the crops [19] could also highlight the advantage. Although the process inevitably introduced extra costs due to the addition of acetate and CO_2_, the significantly enhanced value of this functional starch compared to the normal bulk starch ($30.29 kg (excluding tariffs, provided by Shanxi Green Biotechnology Co., Ltd., Shanxi, China) vs. $0.82/kg [89]) should compensate for and outweigh the costs from the carbon source investment. In fact, acetate may be a promising carbon source for microalgae in terms of economy and sustainability. It is cheaper than glucose (€0.44–0.46/kg vs. €0.55–0.78/kg) and can be produced through different low-carbon processes (e.g., synthesized from C1 gas or liquid/solid wastes) [30]. Further comprehensive techno-economic analysis is needed to confirm the economical feasibility of this strategy.

Although mixotrophic cultivation with acetate addition improved the biomass productivity and starch quality, there remain challenges when scaling up. The main issue in the management of the mixotrophic cultivation of microalgae is contamination control because of the presence of organic carbon (including acetate) [60], which is particularly important for the process with foods as the application products. Photobioreactors should provide a better contamination control, but the capital cost will rise, although this may be offset by the improved algal productivity. From the perspective of sustainability and scalability, the marine microalgae for functional starch production, as was demonstrated herein (*T. subcordiformis*), may be more interesting because of the low demands for fresh water and the lower susceptibility to contamination under the high-salts environment. In addition, the use of acetate in the culture medium may cause additional environmental concerns due to the introduction of extra CODs if it was not consumed up. The recycling of the used medium should be a necessary strategy to maximize the acetate utilization yet with limited rounds since inhibitory effects may occur [90]. Further treatments that eliminate the inhibitors in the medium (such as membrane processes) may be needed before the recycle.

## 4. Conclusions

Both CO_2_ and acetate could be used as carbon sources to promote cell growth, biomass accumulation, and starch production in *Tetraselmis subcordiformis*, with these two types of carbon sources having positive synergistic effects. The addition of an appropriate amount of acetate under CO_2_-enriched conditions could alleviate the oxidative stress, improve the photosynthesis efficiency, and effectively enhance the activity of AGPase, which was conducive to the production of biomass and starch. The supply of CO_2_ and acetate could affect the activities of the starch synthesis-related enzymes (BE and ISA) in *T. subcordiformis* and changed the quality of the starch (Am/Ap ratio). CO_2_ and acetate could also change the morphology and ordered structure of the starch produced, leading to the alteration of digestibility. The combined use of CO_2_ and acetate in the cultivation of *T. subcordiformis* represented the optimal carbon supply strategy to achieve an efficient production of high value-added functional starch (RS). Although the use of acetate for mixotrophic cultivation could face scale-up challenges such as contamination controls and environmental concerns because of the introduction of organics, the sustainable, controllable, and regulable marine microalgae with robust starch accumulation could change the crop-based functional starch manufacturing paradigm in the future. Further research will focus on the elucidation of the correlation of biological mechanism-starch fine structures-starch functionality so that the regulation of RS production in microalgae can be more rational with the help of gene editing (e.g., CRISPR-Cas tools). More robust algal strains with a better tolerance to high-dose acetate and an enhanced RS production ability may be obtained through adaptive laboratory evolution or a synthetic biology approach. Finally, pilot study and full-scale trials are also needed to propel industrial implementation.

## Figures and Tables

**Table 1:** Cultivation conditions under different treatments.

Treatment Symbol	C_CO2_ (%)	C_NaAc_ (g/L)	Time for Starch Characterization
Air-Ac-0	~0.04	0	day 5
Air-Ac-2.5	~0.04	2.5	-
Air-Ac-5	~0.04	5	day 5
Air-Ac-10	~0.04	10	day 5
CO_2_-Ac-0	2	0	day 3
CO_2_-Ac-2.5	2	2.5	day 3
CO_2_-Ac-5	2	5	day 3
CO_2_-Ac-10	2	10	-

**Table 2:** The starch productivity and amylose/amylopectin ratio (Am/Ap) in *Tetraselmis subcordiformis* under different concentrations of acetate (0 g/L, 2.5 g/L, 5 g/L, and 10 g/L) conditions with air (Air-Ac) or 2% CO_2_ (CO_2_-Ac) aerations during the N-deficient cultivation process. The different letters beside the numbers in the same cultivation day represent the significant difference (*p* < 0.05) among various cultivation conditions.

Treatment	Starch Productivity (g/L/day)		Am/Ap	
	Day 3	Day 5	Day 3	Day 5
Air-Ac-0	0.14 ± 0.0 ^ab^	0.17 ± 0.02 ^bc^	0.55 ± 0.01 ^b^	0.49 ± 0.05 ^b^
Air-Ac-2.5	0.20 ± 0.02 ^bc^	0.20 ± 0.02 ^cd^	0.53 ± 0.01 ^b^	0.56 ± 0.02 ^bcd^
Air-Ac-5	0.25 ± 0.02 ^c^	0.24 ± 0.02 ^d^	0.55 ± 0.02 ^b^	0.63 ± 0.03 ^cd^
Air-Ac-10	0.25 ± 0.01 ^c^	0.23 ± 0.02 ^d^	0.60 ± 0.02 ^b^	0.64 ± 0.01 ^d^
CO_2_-Ac-0	0.39 ± 0.01 ^d^	0.15 ± 0.00 ^b^	0.81 ± 0.02 ^c^	0.55 ± 0.03 ^bc^
CO_2_-Ac-2.5	0.71 ± 0.03 ^f^	0.36 ± 0.00 ^e^	0.74 ± 0.01 ^d^	0.57 ± 0.01 ^bcd^
CO_2_-Ac-5	0.59 ± 0.01 ^e^	0.32 ± 0.01 ^e^	0.73 ± 0.02 ^d^	0.50 ± 0.02 ^b^
CO_2_-Ac-10	0.08 ± 0.05 ^a^	0.06 ± 0.03 ^a^	0.45 ± 0.05 ^a^	0.31 ± 0.03 ^a^

**Table 3:** The Am/Ap, particle size, FTIR (R_1047/1022_), and XRD (relative crystallinity, RC) parameter in *Tetraselmis subcordiformis* under different concentrations of acetate (0 g/L, 2.5 g/L, 5 g/L, and 10 g/L) conditions with air (Air-Ac, 5 days) or 2% CO_2_ (CO_2_-Ac, 3 days) aerations during the N-deficient cultivation process. The different letters beside the numbers in the same column represent the significant difference (*p* < 0.05) among various cultivation conditions.

Treatment	Am/Ap	Particle Size (μm)	R_1047/1022_	RC (%)
Air-Ac-0	0.59 ± 0.04 ^a^	1.30 ± 0.32 ^a^	1.24 ± 0.05 ^ab^	26.8 ± 1.1 ^bc^
Air-Ac-5	0.65 ± 0.02 ^ab^	1.51 ± 0.33 ^b^	1.31 ± 0.02 ^b^	29.0 ± 1.4 ^c^
Air-Ac-10	0.70 ± 0.05 ^ab^	1.55 ± 0.48 ^b^	1.29 ± 0.01 ^ab^	27.3 ± 0.3 ^bc^
CO_2_-Ac-0	0.75 ± 0.06 ^b^	1.54 ± 0.35 ^b^	1.26 ± 0.04 ^ab^	25.9 ± 0.9 ^abc^
CO_2_-Ac-2.5	0.72 ± 0.04 ^ab^	1.76 ± 0.33 ^c^	1.24 ± 0.03 ^ab^	24.7 ± 1.4 ^ab^
CO_2_-Ac-5	0.62 ± 0.01 ^ab^	1.57 ± 0.33 ^b^	1.21 ± 0.07 ^a^	22.9 ± 0.6 ^a^

**Table 4:** A comparison of the resistant starch (RS) production in microalgae reported in the literature.

Species	Carbon Source	Nitrogen Source	RS Content (%TS)	RS Content (%DW)	RS Yield (g/L)	References
*Tetraselmis subcordiformis*	Air (0.04% CO_2_)	-N	14.5 ± 2.0	6.3 ± 0.9	0.12 ± 0.02	This study
	Air (0.04% CO_2_) + 5 g/L NaAc	-N	19.4 ± 0.3	9.1 ± 0.3	0.24 ± 0.02	
	Air (0.04% CO_2_) + 10 g/L NaAc	-N	22.4 ± 1.3	10.4 ± 0.8	0.26 ± 0.02	
	2% CO_2_	-N	11.3 ± 0.7	5.9 ± 0.9	0.13 ± 0.01	
	2% CO_2_ + 2.5 g/L NaAc	-N	16.8 ± 1.0	11.1 ± 1.4	0.36 ± 0.04	
	2% CO_2_ + 5 g/L NaAc	-N	22.4 ± 1.2	13.7 ± 0.8	0.40 ± 0.02	
	2% CO_2_ + 12 mM NaHCO_3_	100 mg/L NH_4_^+^-N	55.6	~12.1	0.46	[10]
*Chlorella* sp. MBFJNU-17	40 g/L glucose	4.5 g/L urea	13.0 ± 1.2	~3.4	/	[26]
	15 g/L glucose	-N	15.8 ± 1.4	~8.8	/	[26]
	4 g/L glucose	3 g/L urea	16.4 ± 1.0	~3.4	/	[26]
	40 g/L glucose	1 g/L urea	8.5 ± 1.4	4.7	/	[25]
*Chlorella vulgaris*	1.22 g/L NaAc	0.4 g/L NH_4_Cl	7%	1.1	0.0058	[27]

## Data Availability

The original contributions presented in the study are included in the article/Supplementary Materials, further inquiries can be directed to the corresponding author.

## Supplementary Materials

The following supporting information can be downloaded at https://www.mdpi.com/article/10.3390/foods14112004/s1; Table S1: A comparison of the main components of natural seawater (NSW) and nitrogen-free artificial seawater (ASW-N); Table S2: The concentration of [CH_3_COOH] in *Tetraselmis subcordiformis* under different concentrations of acetate (0 g/L, 2.5 g/L, 5 g/L, and 10 g/L) conditions with air or 2% CO_2_ aerations during the N-deficient cultivation process; Figure S1: Cell growth in *Tetraselmis subcordiformis* under different concentrations of acetate (0 g/L, 2.5 g/L, 5 g/L, and 10 g/L) conditions with air (a) or 2% CO_2_ (b) aerations during the N-deficient cultivation process. The different letters above the column in the same cultivation day represent the significant difference (*p* < 0.05) among various cultivation conditions; Figure S2: The protein (a and b) and oil content (c and d) in *Tetraselmis subcordiformis* under different concentrations of acetate (0 g/L, 2.5 g/L, 5 g/L, and 10 g/L) conditions with air (a and c) or 2% CO_2_ (b and d) aerations during the N-deficient cultivation process. The different letters above the column in the same cultivation day represent the significant difference (*p* < 0.05) among various cultivation conditions; Figure S3: FTIR in *Tetraselmis subcordiformis* under different concentrations of acetate (0 g/L, 2.5 g/L, 5 g/L, and 10 g/L) conditions with air (Air-Ac, 5 days) or 2% CO_2_ (CO_2_-Ac, 3 days) aerations during the N-deficient cultivation process.
