# Supplementary material for: Acetate Combined with CO2 as Effective Carbon Sources for the Production of Resistant Starch in a Marine Microalga Tetraselmis subcordiformis
Source: Foods. 2025 Jun 5;14(11):2004. doi: 10.3390/foods14112004 (PMC12154068; doi:10.3390/foods14112004)
Supplement: Supplementary file 1 [file foods-14-02004-s001.zip › foods-3619905-supplementary.pdf]

Table S1. Comparison of the main components of natural seawater (NSW) and nitrogen-free artificial seawater (ASW-N).

Component	Concentration in NSW (g/L)	Concentration in ASW-N (g/L)
Ca^{2+}	0.41	0.41
Mg^{2+}	1.28	1.34
Na^{+}	10.78	10.62
K^{+}	0.40	0.44
Sr^{2+}	0.008	0
CO_3^{2-}	0.014	0
HCO_3^{-}	0.105	0
SO_4^{2-}	2.71	2.65
Cl^{-}	19.35	18.28
B^{3+}	0.004556	0.0060
F^{-}	0.0013	/
OH^{-}	0.00014	/
PO_4^{2-}+P	0.001721	0.0468
Fe^{3+}	0.0000002077	0.00027
$\sum$	35.17	33.79

Table S2. Concentration of [CH_3COOH] in *Tetraselmis subcordiformis* under different concentrations of acetate (0 g/L, 2.5 g/L, 5 g/L and 10 g/L) conditions with air or 2%CO_2 aerations during the N-deficient cultivation process.

Treatment	c[CH_3COOH](mg/L)	
	day 0	day 1
Air-Ac-2.5	62.65	0.04
Air-Ac-5	78.01	0.06
Air-Ac-10	90.38	0.13
CO_2-Ac-2.5	62.65	7.36
CO_2-Ac-5	78.01	16.89
CO_2-Ac-10	90.38	57.22

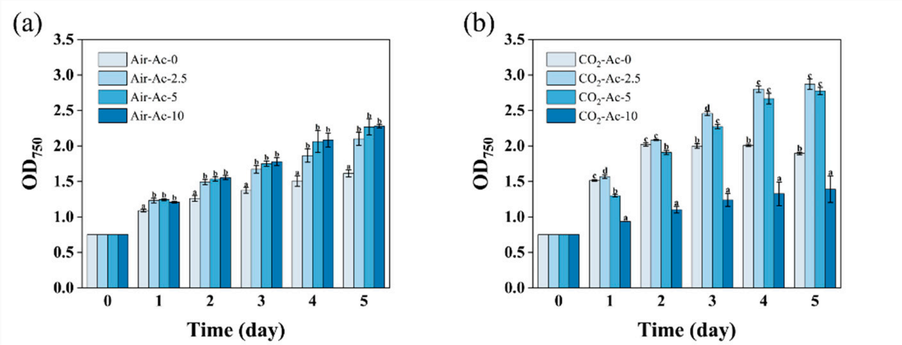

Figure S1. Cell growth (OD_{750}) in *Tetraselmis subcordiformis* under different concentrations of acetate (0 g/L, 2.5 g/L, 5 g/L and 10 g/L) conditions with air (a) or 2%CO_2 (b) aerations during the N-deficient cultivation process. The different letters above the column in the same cultivation day represented significant difference ($p < 0.05$) among various cultivation conditions.

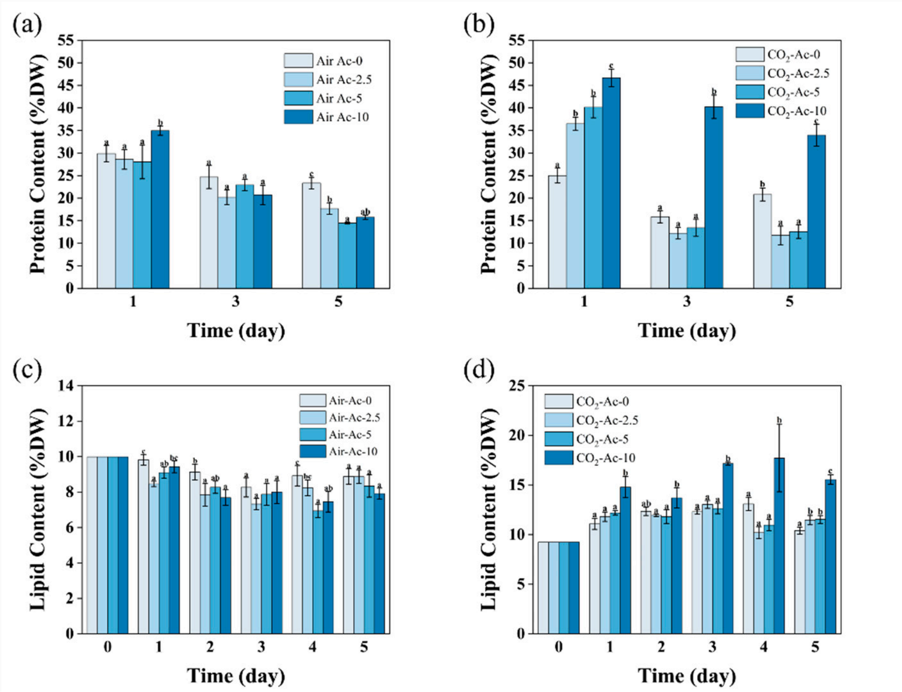

Figure S2. Protein (a and b) and lipid content (c and d) in *Tetraselmis subcordiformis* under different concentrations of acetate (0 g/L, 2.5 g/L, 5 g/L and 10 g/L) conditions with air (a and c) or 2%CO_2 (b and d) aerations during the N-deficient cultivation process. The different letters above the column in the same cultivation day represented significant difference ($p < 0.05$) among various cultivation conditions.

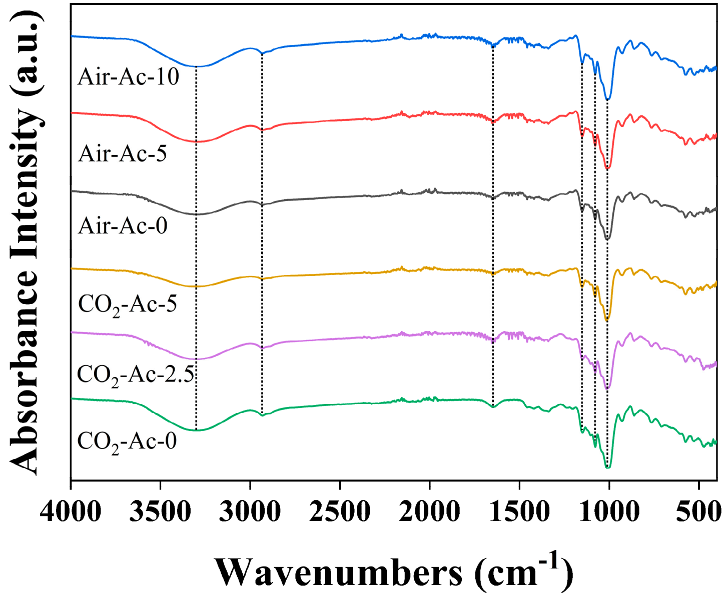

Figure S3. FTIR in *Tetraselmis subcordiformis* under different concentrations of acetate (0 g/L, 2.5 g/L, 5 g/L and 10 g/L) conditions with air or 2%CO_2 aerations during the N-deficient cultivation process.
